# The complete chloroplast genome of *Catalpa fargesii Bur. f. duclouxii (Dode) Gilmour*

**DOI:** 10.1080/23802359.2020.1869606

**Published:** 2021-03-11

**Authors:** Jiwei Sun, Yaqi Li, Xiaotong Ci, Xiyan Li, Dawei Wang, Nianhui Cai, Yulan Xu

**Affiliations:** a Key Laboratory for Forest Resources Conservation and Utilization in the Southwest Mountains of China Ministry of Education, Southwest Forestry University, Kunming, China; b Key Laboratory for Forest Genetic and Tree Improvement and Propagation in Universities of Yunnan Province, Southwest Forestry University, Kunming, China

**Keywords:** *Catalpa fargesii*, chloroplast genome, phylogenetic analysis

## Abstract

In this study, we reported and characterized the complete chloroplast genome sequence of *Catalpa fargesii Bur. f. duclouxii (Dode) Gilmour*. The chloroplast genome was determined to be 158,250 bp in length. It contained large single-copy (LSC) and small single-copy (SSC) regions of 84,929 bp and 12,663 bp, respectively, which were separated by a pair of 30,329 bp inverted repeat (IR) regions. The genome is predicted to contain 121 genes, including 78 protein-coding genes, 35 tRNA genes, and 8 rRNA genes. The overall GC content of the genome is 38.1%. A phylogenetic tree reconstructed by 12 chloroplast genomes reveals that *C. fargesii* is mostly related to *Catalpa. ovata* and *Catalpa. speciosa*. This study identified the unique characteristics of the *C. fargesii* cp genome, which will provide a theoretical basis for species identification and biological research.

*Catalpa fargesii* Bur. f. duclouxii (Dode) Gilmour belongs to the *Catalpa genus* of the Bignoniaceae family and is native to China. *C. fargesii* is distributed within the Yunnan-Guizhou plateau. It is recognized for its straight stems and high-quality timber, which is of high density and has high bending strength and hardness. These characteristics make it a valuable material for furniture production (Xiao et al. 2019). Here, we characterized the complete chloroplast (cp) genome sequence of *C. fargesii*, a complete chloroplast genome sequence from the genus *Catalpa* genus, based on the genome skimming sequencing data. Our data will contribute to our understanding of the genetic resources and evolution of *C. fargesii* based on the diversity in its chloroplast genome and also facilitate the exploration, utilization, and application of conservation genetics of this species.

Chloroplasts are essential organelles in plant cells that play important roles in photosynthesis, carbon fixation, and synthesis of pigments, starch, fatty acids, and amino acids (Daniell et al. 2016). Genome sequencing is frequently used to analyze phylogenetic relationships, genetic diversity, and evolutionary studies (Jose et al. 2015). Three independent genomes offering genetic information are those of the chloroplast, mitochondrion, and nucleus. Compared with the nuclear genome, the chloroplast genome has a small size, single parental inheritance, low nucleotide substitution rate, haploid nature, and highly conserved genomic structure (Wei et al. 2005). Therefore, the chloroplast genome has been considered the perfect model for diversity and evolution studies.

The fresh leaves of *C. fargesii* were collected from Southwest Forestry University Kunming, China. (Yunnan, China; geospatial coordinates: 102°45′41″E, 25°04′00″N). The voucher specimens of *C. fargesii* were deposited at the herbarium of Southwest Forestry University (Voucher number: SWFU-2020-DQ01), and DNA samples were stored at the Key Laboratory for Forest Resources Conservation and Utilization in the Southwest Mountains of China Ministry of Education, Southwest Forestry University, Kunming, China. The total genomic DNA was extracted by using the Magnetic beads plant genomic DNA preps Kit (TSINGKE Biological Technology, Beijing, China). The genome skimming sequencing was conducted on the Illumina HiSeq 2000 Sequencing platform. Genome annotation was assembled with the program Geneious R8 (Biomatters Ltd, Auckland, New Zealand). Finally, the chloroplast DNA sequence with complete annotation information was submitted to GenBank with accession number MW043481.

The complete cp genome of *C. fargesii* is 158,250 bp in length, was 105 bp smaller than that of *Catalpa. ovata* (158,355 bp, MT186670). It was also 11 bp larger than *Catalpa. Speciosa* (158,239 bp, MT319918). The chloroplast genome has the usual quadripartite structure, featuring a LSC region (large single-copy region 84,929 bp), a SSC region (small single-copy region 12,663 bp), and a pair of IR (inverted repeats 30,329 bp). The overall GC content is 38.10% (LSC, 36.43%; SSC, 33.60%; IR, 41.30%). respectively. A total of 121 functional genes were contained in the cp genome, including 78 protein-coding genes, 35 tRNA genes, and 8 rRNA genes.

To determine the phylogenetic location of *C. fargesii* with respect to the other Bignoniaceae with fully sequenced chloroplast genomes, the complete *C. fargesii* chloroplast was used to reconstruct the phylogenetic relationships. With the chloroplast of *Sesamum indicum* (*Sesamum* Pedaliaceae KC569603) and *S. indicum* (*Sesamum* Pedaliaceae JN637766) as an out-group, 12 chloroplast genome sequences of Bignoniaceae, including *Crescentia cujete* (KT182634), *Spathodea campanulata* (MN106255), *Oroxylum indicum* (MN933930), *C. ovata* (MT186670), *C. speciosa* (MT319818), *Podranea ricasoliana* (MG831877), *Tecomaria capensis* (MG831880), *Incarvillea arguta* (MG763885), and *Sesamum indicum* (KC569603), aligned by the MAFFT version 7 programme (Katoh and Standley 2013). A maximum-likelihood analysis based on the GTR + F+R2 model was performed with iqtree version 1.6.7 using 1000 bootstrap replicates (Nguyen et al. 2015). The phylogenetic tree reveals that *C. ovata* and *C. speciosa* is most closely related to *C. fargessi* (Figure 1), the chloroplast genome of *C. fargesii* has enough genetic information to distinguish it from other plants (Wang et al. 2020). This information is crucial for the correct identification of *C. fargesii* and provides valuable genetic resources for the future development of chloroplast derived molecular markers. Our results provide fundamental information for further evolutionary and phylogenetic researches of *C. fargesii*. The chloroplast genome will contribute to the research and conservation of *C. fargesii.*

**Figure 1.**
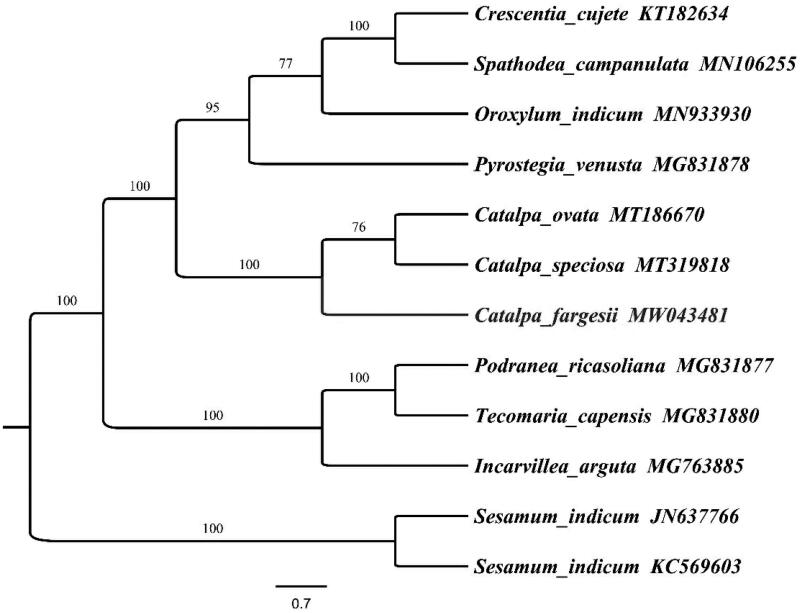
Phylogenetic relationships among 12 complete chloroplast genomes (*C. fargesii* in this study and 11 previously reported species). Bootstrap values based on 1000 replicates were provided near branches. The ML phylogenetic tree for *C. fargesii* based on other 11 species (three in *Catalpa*, one in *Crescentia*, one in *Spathodea*, one in *Oroxylum*, one in *Pyrostegia*, one in *Podranea*, one in *Tecomaria*, one in *Incarvillea*, one in *Sesamum L.*, and one in *Sesamum*) chloroplast genomes.

## Data Availability

The genome sequence data that support the findings of this study are openly available in GenBank of NCBI at (https://www.ncbi.nlm.nih.gov/) under the accession no. MW043481. The associated BioProject, SRA, and Bio-Sample numbers are PRJNA685030, SUB8727632, and SAMN17075338 respectively.

## References

[CIT0001] Daniell H, Lin CS, Yu M, Chang WJ. 2016. Chloroplast genomes: diversity, evolution, and applications in genetic engineering. Genome Biol. 17(1):1–29. 27339192 10.1186/s13059-016-1004-2 PMC4918201

[CIT0002] Jose C-C, Alonso R, Ibañez V, Terol J, Talon M, Dopazo J. 2015. A phylogenetic analysis of 34 chloroplast genomes elucidates the relationships between wild and domestic species within the Genus *Citrus*. Mol Biol Evol. 32(8):2015–2035. 25873589 10.1093/molbev/msv082 PMC4833069

[CIT0004] Katoh K, Standley DM. 2013. MAFFT Multiple sequence alignment software version 7: improvements in performance and usability. Mol Biol Evol. 30(4):772–780. 23329690 10.1093/molbev/mst010 PMC3603318

[CIT0006] Nguyen LT, Schmidt HA, von Haeseler A, Minh BQ. 2015. IQ-TREE: a fast and effective stochastic algorithm for estimating maximum-likelihood phylogenies. Mol Biol Evol. 32(1):268–274. 25371430 10.1093/molbev/msu300 PMC4271533

[CIT0007] Wang Y, Zhao Y, Wang K, Wang L, Feng Y, Qi L, Luo Y, Ji Y, Gong X. 2020. The complete chloroplast genome of *Catalpa ovata* (Bignoniaceae): an important ornamental and medicinal plant. Mitochondrial DNA Part B. 5(2):1675–1676.

[CIT0008] Wei W, Youliang Z, Li C, Yuming W, Zehong Y, Ruiwu Y. 2005. Pcr-rflp analysis of cpdna and mtdna in the genus houttuynia in some areas of china. Hereditas. 142(2005):24–32. 16970608 10.1111/j.1601-5223.2005.01704.x

[CIT0009] Xiao Y, Ma W, Lu N, Wang Z, Wang N, Zhai W, Kong L, Qu G, Wang Q, Wang J. 2019. Genetic variation of growth traits and genotype-by-environment interactions in clones of catalpa bungei and catalpa *fargesii f. duclouxii*. Forests. 10(1):57.

